# Prognostic Value of Pericoronary Adipose Tissue Attenuation After Transcatheter Aortic Valve Replacement in Patients With Aortic Stenosis and Obstructive Coronary Artery Disease

**DOI:** 10.31083/RCM40045

**Published:** 2025-10-31

**Authors:** Tingting Hu, Shuangxiang Lin, Xinfa Ding, Xinhong Wang, Jianzhong Sun

**Affiliations:** ^1^Department of Radiology, The Second Affiliated Hospital Zhejiang University School of Medicine, 310000 Hangzhou, Zhejiang, China

**Keywords:** aortic valve stenosis, coronary artery disease, adipose tissue, transcatheter aortic valve replacement, computed tomography angiography

## Abstract

**Background:**

This study aimed to examine the prognostic value of pericoronary adipose tissue (PCAT) attenuation at three months after transcatheter aortic valve replacement (TAVR) in patients with aortic stenosis (AS) and obstructive coronary artery disease (CAD).

**Methods:**

This retrospective study included 226 patients with both obstructive CAD and AS who underwent TAVR. PCAT attenuation was measured three months post-TAVR using coronary computed tomography angiogram (CCTA) images. Univariable and multivariable Cox regression analyses were conducted to evaluate the association between PCAT attenuation and major adverse cardiac events (MACEs).

**Results:**

Of the 226 patients, 37 experienced MACEs during a median follow-up period of 1.5 years. High PCAT attenuation was significantly associated with MACEs (–65.3 Hounsfield units (HU) vs. –71.6 HU; *p* < 0.01). The optimal PCAT attenuation threshold of –67.5 HU, determined by receiver operating characteristic (ROC) curve analysis, showed 84% sensitivity and 75% specificity (area under the curve (AUC) = 0.88) for predicting MACEs. Multivariable Cox regression confirmed that higher PCAT attenuation was independently associated with an increased risk of MACEs (hazard ratio (HR) = 1.83, 95% confidence interval (CI): 1.44–2.32; *p* < 0.01). Inclusion of PCAT attenuation increased the C-index from 0.41 to 0.82 (*p *= 0.01) and the net reclassification improvement (NRI) by 0.55 (95% CI: 0.34–0.78; *p *= 0.01).

**Conclusions:**

PCAT attenuation was independently associated with the risk of MACEs in post-TAVR patients with obstructive CAD and AS, suggesting the potential utility of PCAT attenuation for risk stratification.

## 1. Introduction

Transcatheter aortic valve replacement (TAVR) has emerged as a transformative intervention in the management of aortic stenosis (AS), significantly improving the symptoms and survival outcomes of elderly patients afflicted by this progressive disease [1]. A notable proportion of individuals with severe AS who undergo TAVR, estimated to range from 60% to 80%, also present with concurrent coronary artery disease (CAD) [2]. This dual pathology is largely driven by shared risk factors such as diabetes, systemic inflammation, gender, and advancing age. The interplay between AS and CAD is characterized by a heightened cardiac workload resulting from AS, which can then exacerbate myocardial ischemia associated with CAD [3, 4]. Conversely, the presence of CAD hampers the heart’s ability to compensate for the increased hemodynamic demands imposed by AS, leading to an accelerated onset of symptoms and the emergence of more severe complications [5].

The use of computed tomography (CT) has become increasingly widespread during the assessment of patients with severe AS for TAVR. CT enhances the diagnostic accuracy and facilitates more effective management strategies [6, 7]. Among the innovations in this area, the analysis of pericoronary adipose tissue (PCAT) has gained attention as a promising non-invasive biomarker for vascular inflammation [8]. PCAT serves as a novel tool for stratifying patients with cardiovascular disease [9]. Extensive research has shown that elevated PCAT attenuation, a marker of increased inflammatory activity, is strongly correlated with a higher risk of major adverse cardiac events (MACE) and worse clinical outcomes [10, 11]. This association highlights the critical involvement of inflammatory mediators, such as interleukins and tumor necrosis factor-alpha, in the processes of tissue remodeling, calcification, and plaque instability, all of which contribute to cardiovascular risk [12]. The role of PCAT in mediating these inflammatory pathways underscores its potential utility as a prognostic marker.

Based on prior literature, we hypothesized that PCAT analysis of the culprit vessel or of the post-stent implantation vessel subsequent to TAVR could be instrumental in identifying patients at high risk for MACE. Therefore, the aim of this study was to evaluate the prognostic significance of coronary PCAT measured three months post-TAVR in predicting cardiovascular events among CAD patients.

## 2. Materials and Methods

### 2.1 Study Population

**Fig. 1.**
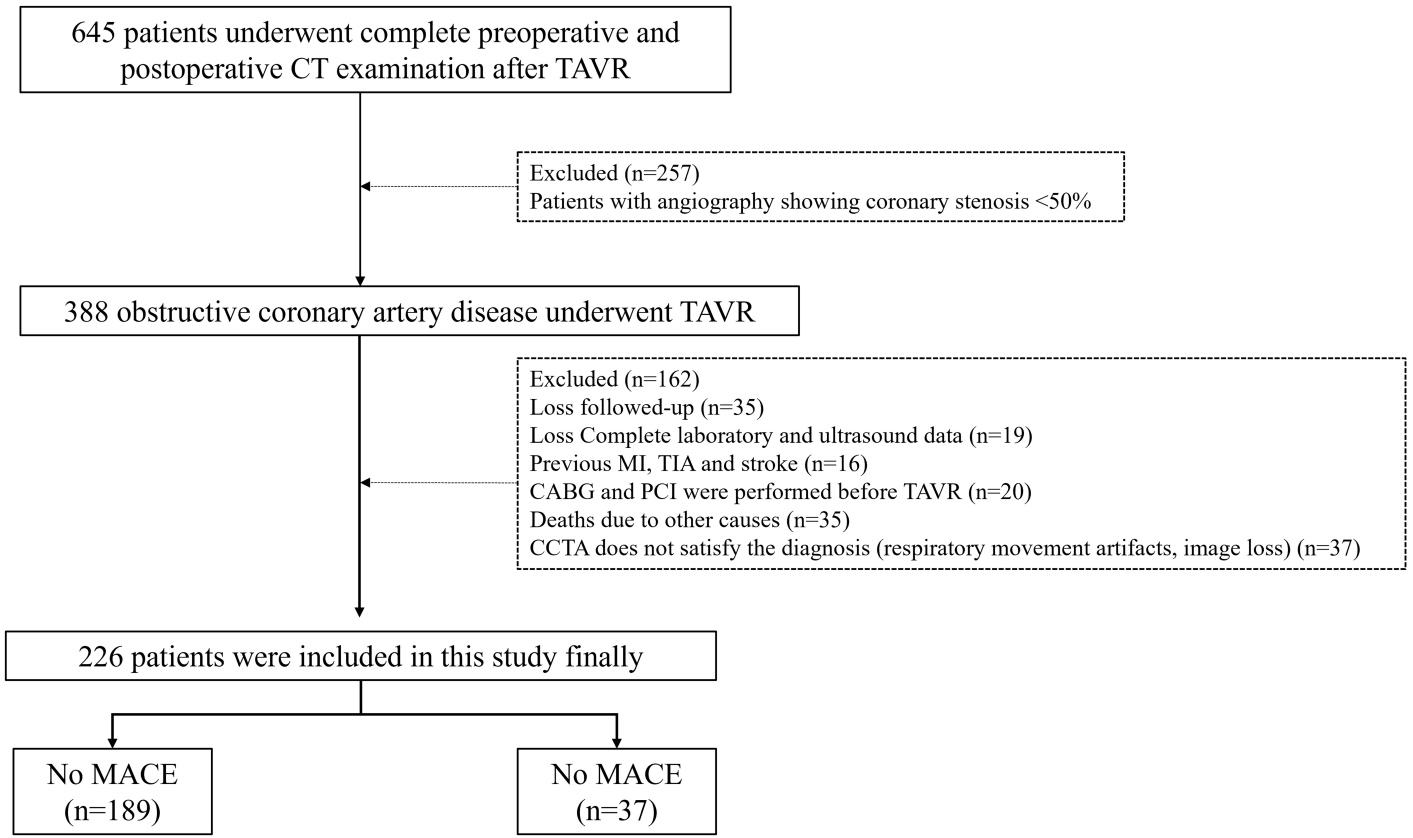
**Flowchart of study population**. CT, computed tomography; TAVR, transcatheter aortic valve replacement; MI, Myocardial infarction; TIA, Transient ischemic attack; CABG, Coronary artery bypass; CCTA, coronary computed tomography angiogram; MACE, major adverse cardiac events.

This retrospective study included consecutive patients with severe AS and obstructive CAD, defined as at least one stenosis ≥50% in a major epicardial coronary artery on invasive angiography. The patients underwent TAVR at our institution between January 2018 and September 2023, followed by post-TAVR coronary CT angiography (CCTA) approximately 3 months after the procedure. All patients had baseline diagnostic coronary angiography and were considered for revascularization by a heart team. Furthermore, all diseased blood vessels underwent percutaneous coronary intervention (PCI) before the TAVR procedure [13]. Exclusion criteria were: (1) non-obstructive CAD (coronary stenosis <50% on angiography); (2) incomplete key clinical data (laboratory or echocardiographic measurements) required for the study; (3) history of myocardial infarction, transient ischemic attack, or stroke prior to TAVR (as these could confound the outcomes); (4) prior coronary artery bypass grafting or valve surgery (since these could affect both the procedure and outcomes); (5) death due to causes unrelated to cardiovascular disease during follow-up (thus allowing a focus on CV outcomes); (6) lost to follow-up (i.e., no outcome data was obtained after the initial 3-month post-TAVR CT scan and up to the end of the study period, or incomplete data); and (7) CCTA images of insufficient quality for PCAT analysis (due to artifacts or data loss). The flow chart for patient selection is shown in Fig. 1.

### 2.2 CT Scanning Techniques

Patients underwent CCTA using a third-generation dual-source CT scanner (Somatom Force; Siemens Healthineers, Forchheim, Germany) in dual-energy mode. The X-ray tubes were set at 80 kV (387 mAs) and Sn150 kV (215 mAs with a 0.64-mm tin filter). The scan parameters included a detector collimation of 192 × 0.6 mm, rotation time of 0.25 seconds, slice thickness of 0.75 mm, increment of 0.5 mm, ADMIRE strength level 4, Kernel Bv36, and a temporal resolution of 66 ms. The protocol spanned from the thoracic entrance to the base of the diaphragm using retrospective electrocardiogram gating. For contrast, 50–65 mL of iodinated contrast medium (Ultravist 370 mg I/mL, Bayer Schering Pharma) was injected intravenously at 4 to 5 mL/sec, followed by a saline flush at the same rate. Scans commenced when the contrast density in the ascending aorta reached 210 Hounsfield units (HU). Images were then processed at a dedicated workstation (Syngo.via, version VB40, Siemens Healthineers, Forchheim, Germany).

### 2.3 Analysis of Pericoronary Adipose Tissue 

As previously described in the literature, PCAT analysis was performed on a specialized workstation (Cardiac Risk Assessment Prototype, Syngo.via Frontier, Siemens Healthineers) and was based on 80 kV-generated images [14, 15]. Narrowed coronary vessels were analyzed, and in the case of multi-vessel disease, vessels with the most severe stenosis were selected. A 40 mm segment of the proximal coronary vessel for the stenotic vessel (10 mm to 50 mm from its origin) was selected to avoid influence from the aortic wall. Vessel lumen and wall boundaries were initially identified automatically, then adjusted manually if necessary. Perivascular adipose tissue was quantified as voxels, with attenuation values between –190 and –30 HU. PCAT attenuation was calculated as the mean CT attenuation, adjusted for technical factors [16]. Two blinded observers interpreted the images after a half-day interval to ensure objectivity.

### 2.4 Outcomes

Follow-up assessments were conducted quarterly until March 2024 through telephone interview and review of medical records. MACE were defined as the composite of cardiovascular death, non-fatal myocardial infarction, cardiac arrest, ischemic stroke of cardiac origin, or acute coronary syndrome requiring unplanned revascularization

### 2.5 Statistical Analysis

Baseline characteristics for categorical variables are presented as rates and percentages, and for continuous variables as medians with interquartile ranges (IQR). Chi-square tests and Student’s *t*-tests were used to analyze categorical and continuous data, respectively. ROC curve analysis was performed to determine optimal cutoff values using the Youden index. Survival was estimated with the Kaplan-Meier proportional hazard’s method, and differences assessed using the stratified log-rank test. Univariable Cox regression analysis was used to identify clinical factors associated with MACE. A multivariable Cox model was constructed to assess the independent association of PCAT with MACE. Clinical covariates associated with MACE were included using a stepwise forward selection algorithm that retained variables with a *p*-value < 0.05. PCAT was then added to the multivariable model to determine its incremental prognostic value. The change in model discrimination with the addition of PCAT was calculated using Harrell’s c-statistic. Since the clinical risk categories related to PCAT treatment are not well defined, we assessed the net reclassification improvement (NRI).

## 3. Results

### 3.1 Patient Characteristics

The study cohort comprised 226 obstructive CAD patients who underwent TAVR. These were predominantly male (59.5%) and had a mean age of 72.6 years (IQR, 68–78.8). During a median follow-up period of 1.5 years (IQR, 0.7–2.4 years), 37 of the 226 participants (16.4%) experienced MACE. Table 1 shows the patient characteristics for the overall cohort, as well as for the subgroups with and without MACE. Of the 678 analyzed vessels, the right coronary artery was the most frequently implicated vessel (70.4%). However, it was not significantly associated with MACE. Patients who experienced MACE were older (median age 78.0 vs. 72.0, *p* < 0.001) and had lower BMI (21.6 vs. 23.1, *p* = 0.008). Furthermore, laboratory results indicated that MACE patients were more likely to be anemic (119.0 g/L vs. 131.0 g/L, *p* = 0.004) and have elevated Pro-BNP (2970 pg/mL vs. 1262 pg/mL, *p* = 0.01). Echocardiography revealed that MACE patients had a smaller aortic valve area (0.6 cm^2^ vs. 0.7 cm^2^, *p* = 0.019). No significant differences in post-TAVR procedural outcomes (paravalvular leak and pacemaker implantation) were observed between the two groups. CT imaging also revealed a higher incidence of calcified valves (67.6% vs. 46.6%, *p* = 0.031) in the MACE group.

**Table 1.** **Patient characteristics and imaging findings in patients with or without MACE**.

Characteristic		Overall	No MACE	MACE	*p*-value
		(n = 226)	(n = 189)	(n = 37)	
Age (years)		72.6 (68.0, 78.8)	72.0 (68.0, 76.0)	78.0 (72.0, 82.0)	< **0.01**
Gender (male)		134 (59.3)	110 (58.2)	24 (64.9)	0.45
BMI (kg/m^2^)		22.9 (3.2)	23.1 (3.2)	21.6 (3.0)	**0.01**
BSA (m^2^)		1.6 (1.5, 1.7)	1.6 (1.5, 1.8)	1.6 (1.5, 1.6)	**0.02**
Smoking (Yes)		61 (27.0)	49 (25.9)	12 (32.4)	0.42
Dyslipidemia (Yes)		27 (12.0)	24 (12.8)	3 (8.1)	0.61
Hypertension (Yes)		118 (52.2)	99 (52.4)	19 (51.4)	0.91
Diabetes (Yes)		41 (18.1)	34 (18.0)	7 (18.9)	0.89
Angina (Yes)		95 (42.0)	84 (44.4)	11 (29.7)	0.11
Syncope (Yes)		15 (6.7)	14 (7.4)	1 (2.7)	0.49
NYHA III		3.0 (2.0, 3.0)	3.0 (2.0, 3.0)	3.0 (3.0, 4.0)	< **0.01**
STS score		3.2 (1.9, 5.5)	2.9 (1.8, 4.8)	5.4 (3.2, 8.4)	**0.01**
Laboratory					
	Leukocytes (10^9^/L)	6.1 (4.8, 7.3)	6.0 (4.8, 7.3)	6.6 (5.1, 7.4)	0.32
	Hemoglobin (g/L)	130.0 (117.0, 140.1)	131.0 (119.0, 141.0)	119.0 (102.0, 137.0)	**0.01**
	Platelet count (10^9^/L)	173.5 (144.0, 204.8)	171.0 (144.0, 204.0)	179.0 (156.0, 205.0)	0.46
	ProBNP (pg/mL)	1377 (430.0, 4594.1)	1262 (295.6, 4022.3)	2970.0 (760.0, 7108.0)	**0.01**
	TnT (ng/mL)	0.05 (0.12)	0.05 (0.13)	0.05 (0.05)	0.89
Echocardiography					
	Left atrial diameter (cm)	4.2 (3.9, 4.6)	4.2 (3.9, 4.6)	4.2 (3.8, 4.4)	0.12
	Left ventricular ejection fraction (%)	59.1 (48.0, 64.9)	59.1 (49.2, 64.7)	56.8 (46.6, 64.9)	0.94
	Max velocity (m/s)	4.5 (4.0, 5.2)	4.4 (3.8, 5.2)	4.6 (4.1, 5.2)	0.22
	Mean gradient (mmHg)	46.0 (37.0, 62.0)	46.0 (36.7, 61.0)	51.0 (39.0, 67.0)	0.16
	Aortic valve area (cm^2^)	0.7 (0.5, 0.9)	0.7 (0.6, 0.9)	0.6 (0.5, 0.8)	**0.02**
Valve Type					0.51
	II	109 (48.2)	93 (49.2)	16 (43.2)	
	III	117 (51.8)	96 (50.8)	21 (56.8)	
STJ av. diameter (mm)		30.8 (27.9, 33.6)	31.0 (28.1, 34.0)	30.3 (27.3, 32.8)	0.09
STJ height (mm)		21.4 (19.1, 24.1)	21.6 (19.1, 24.2)	20.8 (19.1, 22.6)	0.05
Max ascend. aorta diameter (mm)		39.7	40.1	38.1	0.38
		(36.2, 43.7)	(36.6, 43.6)	(35.3, 45.3)	
Calcified		113 (50.0)	88 (46.6)	25 (67.6)	**0.03**
PCAT (HU)		–69.9	–71.6	–65.3	< **0.01**
		(–74.6, –66.6)	(–75.5, –67.6)	(–67.0, –63.1)	
Coronary artery disease location					
	RCA	159 (70.4)	133 (70.4)	26 (70.3)	1.00
	LCX	39 (17.3)	33 (17.5)	6 (16.2)	0.86
	LAD	28 (12.4)	23 (12.2)	5 (13.5)	0.99
	PCI	116 (51.3)	100 (52.9)	16 (43.2)	0.28
Post TAVR					
	Paravalvular leak	28 (12.39)	23 (12.17)	5 (13.51)	0.18
	Pacemaker implantation	26 (11.51)	20 (10.69)	6 (16.22)	0.06

Values are either n (%) or median (IQR). Statistically significant *p*-values are in bold. BMI, body mass index; BSA, body surface area; NYHA, New York Heart Association; STS score, Society of Thoracic Surgeons Score; Pro-BNP, Pro-B-type Natriuretic Peptide; STJ, sinotubular junction; PCAT, Pericoronary adipose tissue; RCA, right coronary artery; LCX, left circumflex; LAD, left anterior descending; PCI, percutaneous coronary intervention; HU, Hounsfield units.

### 3.2 Findings for Pericoronary Adipose Tissue 

**Fig. 2.**
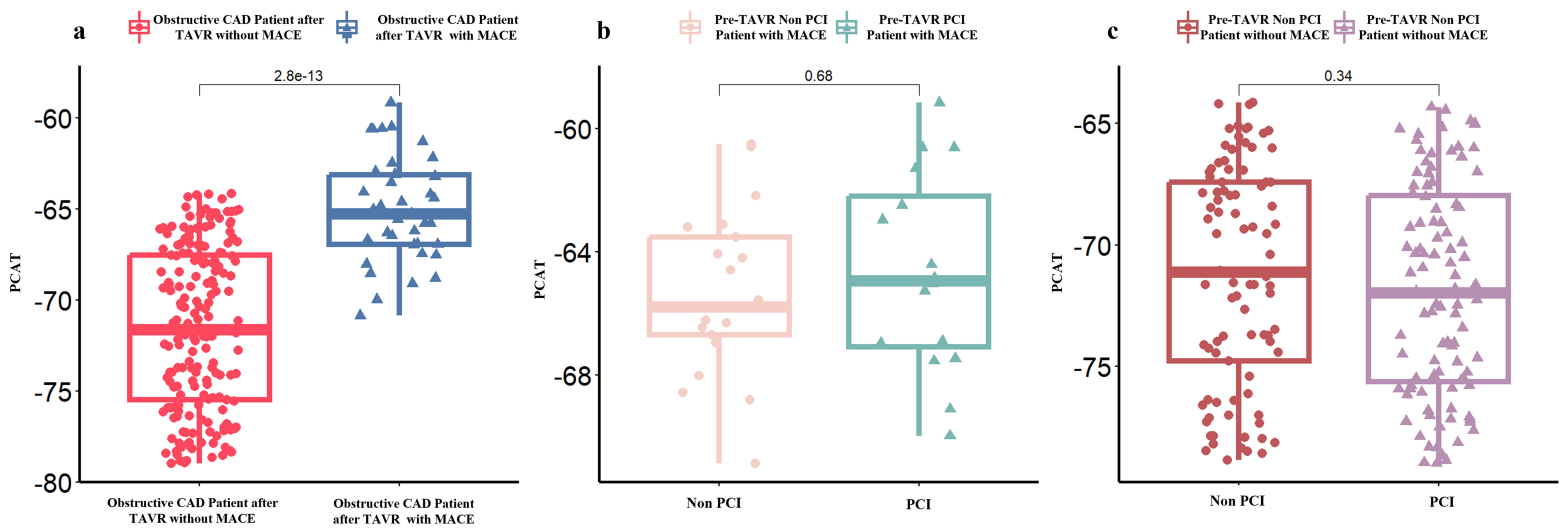
**Pericoronary Adipose Tissue (PCAT) attenuation in obstructive CAD patients post-TAVR**. (a) Obstructive CAD patients after TAVR with or without major adverse cardiac events (MACE). (b) Whether to perform PCI in Obstructive CAD Patient after TAVR with MACE, and (c) Whether to perform PCI in Obstructive CAD Patient after TAVR without MACE.

**Fig. 3.**
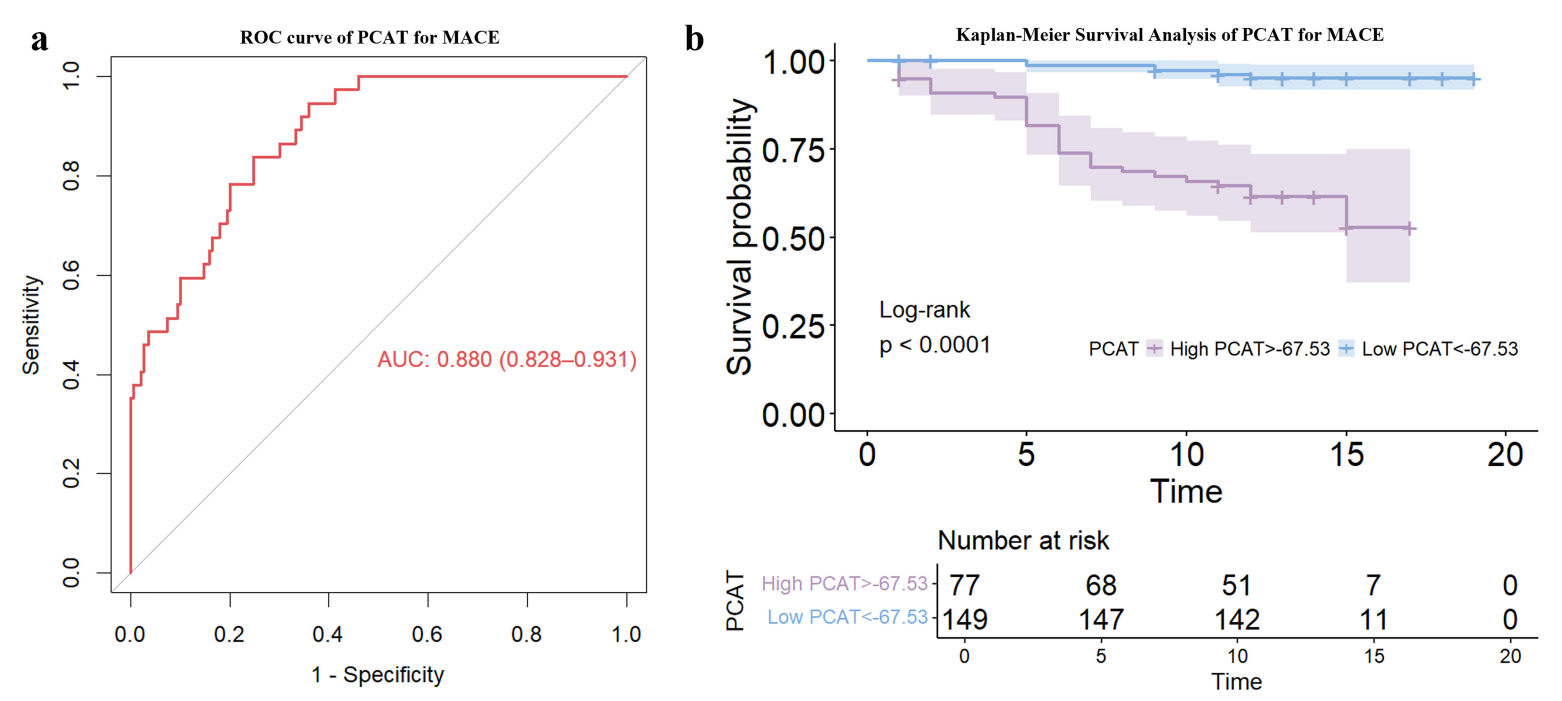
**Evaluation of PCAT attenuation as a prognostic indicator in obstructive CAD patients post-TAVR**. (a) Receiver Operating Characteristic (ROC) curve. (b) Kaplan-Meier survival analysis.

As shown in Fig. 2a, patients who experienced MACE following TAVR showed higher PCAT attenuation compared to those without MACE (–65.3 HU vs. –71.6 HU, *p* < 0.001). However, no significant difference in PCAT attenuation was observed between pre-TAVR patients with MACE who did or did not undergo PCI (Fig. 2b), and similarly for patients without MACE (Fig. 2c). ROC curve analysis identified an optimal PCAT attenuation threshold of –67.5 HU for the prediction of MACE with 84% sensitivity, 75% specificity, and an area under the curve (AUC) of 0.88 (Fig. 3a). Kaplan-Meier survival analysis revealed a significant association between PCAT attenuation and an increased risk of adverse events (Fig. 3b). Table 2 shows the clinical and laboratory characteristics of high and low PCAT groups defined according to the threshold value of –67.5 HU. Significantly higher ProBNP levels were observed in the low PCAT group (5315.46 ± 8371.55 vs. 3293.08 ± 4909.42, *p* = 0.04). A greater proportion of patients in the low PCAT group experienced a worse status (38.96% vs. 4.70%, *p* < 0.01). Furthermore, the follow-up duration was significantly shorter in the low PCAT group (9.86 ± 4.24 months vs. 12.33 ± 2.32 months, *p* < 0.01). Pacemaker implantation prevalence differed significantly between groups (High PCAT: 12.75% vs. Low PCAT: 9.10%, *p* = 0.03). No significant differences between the high and low PCAT groups were found for other clinical characteristics, laboratory results, or echocardiographic measurements.

**Table 2.** **Clinical and laboratory characteristics associated with high and low Pericoronary Adipose Tissue (PCAT) attenuation**.

Characteristic		Overall	High PCAT	Low PCAT	*p*-value
		(n = 226)	(n = 149)	(n = 77)	
Age (years)		73.00 ± 7.78	72.54 ± 7.48	73.88 ± 8.29	0.22
Gender (male)		134 (59.29)	88 (59.06)	46 (59.74)	0.92
BMI (kg/m^2^)		22.89 ± 3.25	23.07 ± 3.26	22.55 ± 3.21	0.25
BSA (m^2^)		1.63 ± 0.17	1.64 ± 0.18	1.62 ± 0.17	0.37
Smoking (Yes)		61 (26.99)	42 (28.19)	19 (24.68)	0.57
Dyslipidemia (Yes)		27 (11.95)	18 (12.08)	9 (11.69)	0.93
Hypertension (Yes)		118 (52.21)	77 (51.68)	41 (53.25)	0.82
Diabetes (Yes)		41 (18.14)	29 (19.46)	12 (15.58)	0.47
Angina (Yes)		95 (42.04)	65 (43.62)	30 (38.96)	0.50
Syncope (Yes)		15 (6.64)	13 (8.72)	2 (2.60)	0.08
NYHA III		195 (86.28)	131 (87.92)	64 (83.12)	0.42
STS score		4.31 ± 3.69	3.93 ± 2.96	5.04 ± 4.74	0.06
Laboratory					
	Leukocyte (10^9^/L)	6.22 ± 2.14	6.22 ± 2.21	6.21 ± 2.00	0.95
	Hemoglobin (g/L)	127.47 ± 20.48	129.11 ± 19.99	124.32 ± 21.19	0.10
	Platelet count (10^9^/L)	178.35 ± 58.32	179.44 ± 54.68	176.24 ± 65.10	0.70
	ProBNP (pg/mL)	3982.12 ± 6359.97	3293.08 ± 4909.42	5315.46 ± 8371.55	**0.04**
	TnT (ng/mL)	0.05 ± 0.12	0.05 ± 0.08	0.06 ± 0.17	0.37
Echocardiography					
	Left atrial diameter (cm)	4.24 ± 0.58	4.26 ± 0.60	4.20 ± 0.52	0.49
	Left ventricular ejection fraction (%)	56.45 ± 11.68	56.39 ± 12.06	56.57 ± 10.99	0.91
	Max velocity (m/s)		4.43 ± 1.22	4.25 ± 1.27	0.29
	Mean gradient (mmHg)	48.34 ± 24.03	49.36 ± 23.59	46.37 ± 24.89	0.38
	Aortic valve area (cm^2^)	0.82 ± 0.50	0.82 ± 0.54	0.81 ± 0.44	0.87
Valve Type					0.38
	II	109 (48.23)	75 (50.34)	34 (44.16)	
	III	117 (51.77)	74 (49.66)	43 (55.84)	
STJ average diameter (mm)		31.36 ± 4.72	31.50 ± 4.80	31.08 ± 4.59	0.53
STJ height (mm)		22.24 ± 5.10	22.12 ± 4.33	22.47 ± 6.36	0.63
Max ascend. aorta diameter (mm)		40.23 ± 6.53	40.31 ± 6.15	40.08 ± 7.25	0.80
Calcified valve		169 (74.78)	106 (71.14)	63 (81.82)	0.08
Coronary artery disease location					
	RCA	28 (12.39)	20 (13.42)	8 (10.39)	0.51
	LCX	39 (17.26)	25 (16.78)	14 (18.18)	0.79
	LAD	159 (70.35)	104 (69.80)	55 (71.43)	0.80
	PCI	116 (51.33)	81 (54.36)	35 (45.45)	0.20
Post-TAVR					
	Paravalvular leak	28 (12.39)	20 (13.42)	8 (10.39)	0.55
	Pacemaker implantation	26 (11.51)	19 (12.75)	7 (9.10)	0.03
	Patient experienced MACE	37 (16.37)	7 (4.70)	30 (38.96)	< **0.01**
	Follow-up time (months)	11.49 ± 3.31	12.33 ± 2.32	9.86 ± 4.24	< **0.01**

Values are either n (%) or median (IQR). Statistically significant *p*-values are printed in bold. BMI, body mass index; BSA, body surface area; NYHA, New York Heart Association; STS score, Society of Thoracic Surgeons Score; Pro-BNP, Pro-B-type Natriuretic Peptide; STJ, sinotubular junction; PCAT, Pericoronary adipose tissue; RCA, right coronary artery; LCX, left circumflex; LAD, left anterior descending; PCI, percutaneous coronary intervention.

### 3.3 Association of Clinical Characteristics With Pericoronary Adipose Tissue

**Fig. 4.**
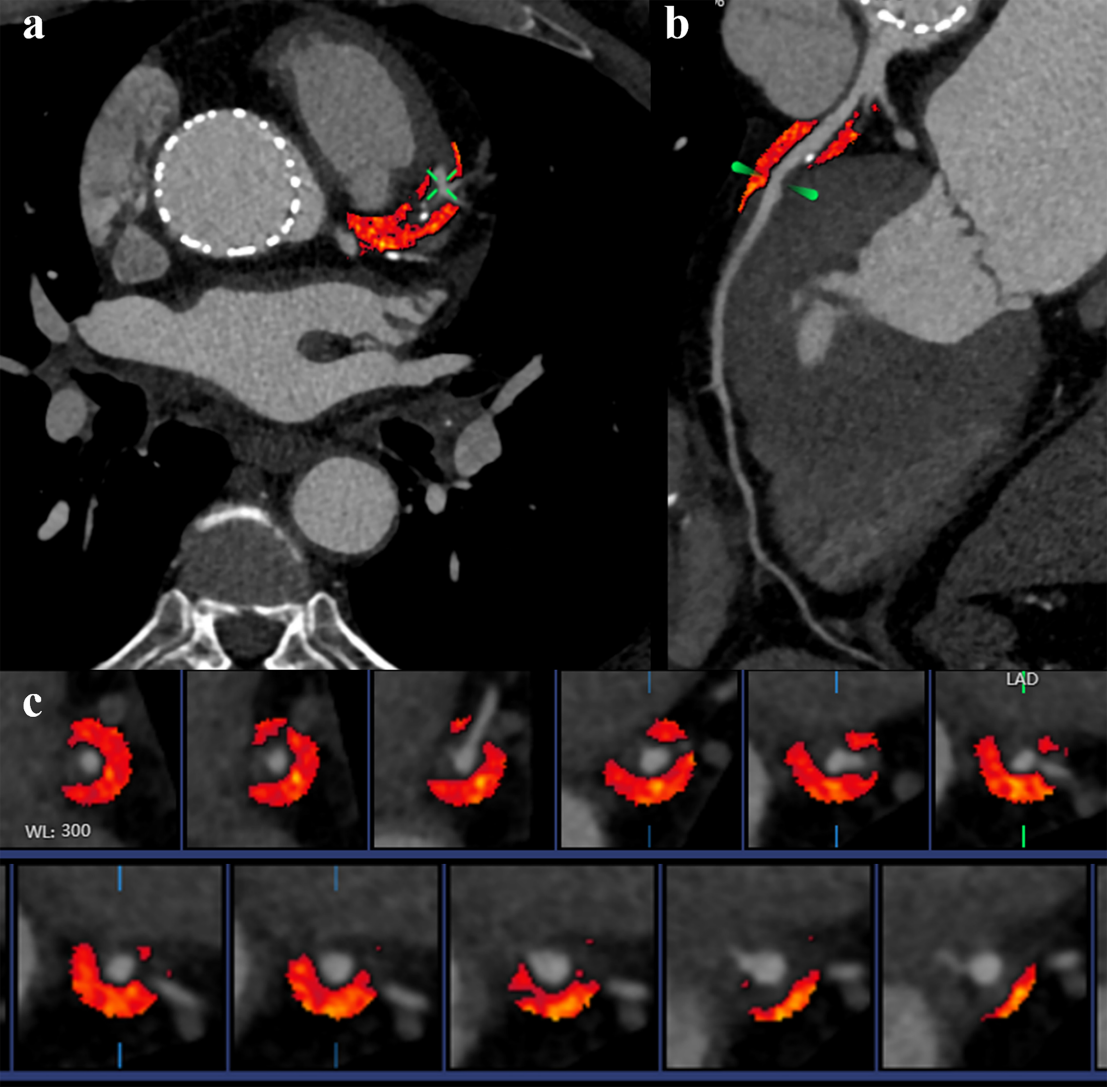
**Representative examples of Pericoronary Adipose Tissue (PCAT) attenuation**. The CCTA image from a 75-year-old male patient 3 months after TAVR. (a) PCAT surrounding left anterior descending in axial plane. (b) Curved reconstruction of the left anterior descending. (c) PCAT around left anterior descending (red area).

Table 3 shows the association of various clinical factors with MACE. In univariate analysis, the characteristics of patient age, NYHA functional class, angina, hemoglobin level, aortic valve area, sinotubular junction (STJ) height, STJ diameter, valve calcification and PCAT attenuation were all significantly associated with MACE. Following adjustment for confounding factors, multivariate Cox regression analysis revealed that Angina, Aortic valve area, NYHA functional class, STJ diameter, STJ height, valve calcification and PCAT attenuation remained significant predictors of MACE. Fig. 4 presents the measurement results of PCAT for a patient.

**Table 3.** **Univariable and multivariable Cox regression analysis of the association of clinical characteristics and imaging findings with MACE**.

Clinical characteristics		Univariable		Multivariable	
		Hazard ratio (95% CI)	*p*-value	Hazard ratio (95% CI)	*p*-value
Age (years)		1.07 (1.02, 1.12)	0.01	1.06 (0.98, 1.15)	0.17
Gender (male)		1.14 (0.55, 2.36)	0.72	-	-
BMI (kg/m^2^)		0.90 (0.81, 1.01)	0.07	-	-
BSA (m^2^)		0.15(0.02, 1.18)	0.07	-	-
Smoking (Yes)		1.30 (0.61, 2.75)	0.49	-	-
Dyslipidemia (Yes)		1.22 (0.37, 4.05)	0.74	-	-
Hypertension (Yes)		0.66 (0.32, 1.38)	0.27	-	-
Diabetes (Yes)		1.11 (0.47, 2.58)	0.82	-	-
Angina (Yes)		0.53 (0.25, 1.13)	0.04	0.95 (0.92, 1.99)	<0.01
Syncope (Yes)		0.77 (0.10, 5.66)	0.80	-	-
NYHA		2.25 (1.30, 3.91)	<0.01	4.53 (1.34, 8.38)	0.02
STS score		1.06 (1.00, 1.12)	0.06	-	-
Laboratory					
	Leukocyte (10^9^/L)	1.04 (0.90, 1.20)	0.62		
	Hemoglobin (g/L)	0.97 (0.36, 1.36)	<0.01	0.98 (0.95, 1.01)	0.07
	Platelet count (10^9^/L)	1.00 (1.00, 1.01)	0.37	-	-
	ProBNP (pg/mL)	1.00 (1.00, 1.00)	0.29	-	-
	TnT (ng/mL)	0.57 (0.10, 2.29)	0.52	-	-
Echocardiography					
	Left atrial diameter (cm)	0.79 (0.43, 1.45)	0.45	-	-
	Left ventricular ejection fraction (%)	1.01 (0.98, 1.04)	0.72	-	-
	Max velocity (m/s)	1.35 (0.99, 1.85)	0.06	-	-
	Mean gradient (mmHg)	1.01 (1.00, 1.03)	0.11	-	-
	Aortic valve area (cm^2^)	0.11 (0.02, 0.58)	0.01	0.18 (0.01, 0.95)	0.04
	Valve type	1.20 (0.59, 2.44)	0.61	-	-
	STJ average diameter (mm)	0.88 (0.81, 0.97)	<0.01	0.92 (0.84, 1.92)	0.02
	STJ height (mm)	0.88 (0.79, 0.98)	0.02	1.01 (0.87, 2.17)	0.01
	Max ascend. aorta diameter (mm)	1.05 (0.93, 1.19)	0.41	-	-
	Calcified valve	2.74 (1.27, 5.90)	0.01	1.78 (1.14, 3.25)	0.04
PCAT		1.46 (1.30, 1.64)	<0.01	1.83 (1.44, 2.32)	<0.01
Coronary artery disease location					
	LAD	1.60 (0.71, 3.61)	0.26	-	-
	LCX	0.31 (0.09, 1.05)	0.06	-	-
	RCA	1.49 (0.57, 3.91)	0.41	-	-
	PCI	0.92 (0.45, 1.86)	0.81	-	-
Post TAVR					
	Paravalvular leak	1.62 (0.79, 3.31)	0.18	-	-
	Pacemaker implantation	1.30 (0.63, 2.65)	0.47	-	-

BMI, body mass index; BSA, body surface area; NYHA, New York Heart Association class; STS score, Society of Thoracic Surgeons Score; Pro-BNP, Pro-B-type Natriuretic Peptide; STJ, sinotubular junction; PCAT, Pericoronary adipose tissue; RCA, right coronary artery; LCX, left circumflex; LAD, left anterior descending; PCI, percutaneous coronary intervention.

Statistical modeling results shown in Table 4 indicate that Model 2 represents a significant improvement over Model 1, with a C-index of 0.41 (*p* = 0.03) and an NRI of 0.24 (*p* = 0.02). Furthermore, Model 3 exhibited a marked enhancement in both the C-index (0.82, *p* = 0.01) and NRI (0.55, *p* = 0.01).

**Table 4.** **Multivariable analysis of patient characteristics and imaging findings for the prediction of MACE**.

Model	C-index (95% CI)	*p*-value	NRI (95% CI)	*p*-value
1	0.23 (0.11, 0.37)	-	-	-
2	0.41 (0.23, 0.57)	0.03	0.24 (0.15, 0.29)	0.02
3	0.82 (0.68, 0.95)	0.01	0.55 (0.34, 0.78)	0.01

Model 1: Traditional clinical risk model (Angina + NYHA + Aortic valve area + STJ average diameter). Model 2: Traditional clinical risk model + Calcified valve. Model 3: Traditional clinical risk model + Calcified + PCAT. NRI, Net reclassification improvement; PCAT, Pericoronary adipose tissue; NYHA, New York Heart Association class.

## 4. Discussion

This study found that measurement of PCAT attenuation by CCTA three months after TAVR could effectively predict MACE in patients with obstructive CAD. The key finding was that high PCAT attenuation was associated with a significantly increased risk of MACE, with a cutoff threshold of –67.5 HU demonstrating high sensitivity and specificity for predicting adverse outcomes. These results highlight the potential of PCAT attenuation appears to be a promising marker for risk stratification in this high-risk patient population.

The risk factors for AS overlap significantly with those for atherosclerosis, explaining why >50% of patients with severe symptomatic AS also present with concurrent CAD. However, the management of CAD in the context of TAVR remains challenging due to the lack of large randomized controlled trials (RCTs) and the exclusion of patients with significant CAD in previous studies on TAVR [17]. Steyer *et al*. [18] previously reported that PCAT attenuation in the RCA, measured pre-TAVR, was a significant predictor of outcomes in AS patients undergoing TAVR [19]. Our study differs in that we assessed PCAT attenuation post-TAVR and evaluated the most diseased coronary segment, rather than focusing exclusively on the RCA. Contrary to previous reports, we did not find that PCAT attenuation in any specific coronary artery was singularly predictive of MACE. This observation aligns with the findings of Meng *et al*. [20] and Napoli *et al*. [21] who reported that higher PCAT attenuation was associated with adverse outcomes in CAD patients, including myocardial infarction and cardiovascular mortality. However, our study advances the field by specifically addressing TAVR patients, a subgroup with both AS and CAD. These patients present a unique challenge in risk stratification. Our results suggest that PCAT attenuation could prove valuable in determining the prognosis of post-TAVR patients, thereby improving risk prediction. Furthermore, when integrated into multivariable models, PCAT attenuation enhanced the prognostic value of traditional clinical and imaging markers, such as valve calcification and NYHA functional class, thus offering incremental predictive value over established markers. This underscores the potential of PCAT attenuation as a non-invasive and robust biomarker in the post-TAVR setting, which may contribute to better patient management and outcomes.

PCAT has emerged as a promising biomarker for assessing coronary inflammation and plaque stability [22]. With the increasing incidence of AS and subsequent rise in TAVR procedures, the need for reliable markers to predict postoperative outcomes in CAD patients is becoming more critical. The utility of PCAT in this context has been substantiated by numerous studies, including the Comprehensive Risk Prediction in Surgery (CRISP-CT) study, which underscores the ability of PCAT attenuation to predict mortality risk [23]. Furthermore, Elnabawi *et al*. [24] highlighted the versatility of PCAT by demonstrating its effectiveness in tracking therapeutic responses in CAD, thus expanding its application beyond mere prognostication [25].

The role of PCAT has assumed greater significance in the post-TAVR landscape, with the procedure itself capable of inducing biological and physical stresses on the coronary vessels. This is particularly evident in the hemodynamic changes brought about by the newly implanted aortic valve, which directly influence myocardial oxygen supply and demand [26]. In the case of a prosthesis-patient mismatch or suboptimal deployment of the valve, the anticipated alleviation of myocardial ischemia may not be fully realized. Such instances can lead to inadequate coronary perfusion, especially in patients with existing CAD [27]. Moreover, the postoperative phase can invoke systemic inflammatory responses, potentially accelerating the progression of atherosclerosis and contributing to plaque vulnerability [28, 29]. Collectively, these factors have significant implications for the long-term cardiac prognosis of CAD patients post-TAVR.

Currently, there is significant debate regarding prognostic markers for CAD combined with AS after TAVR. Examples of such markers include the Duke Myocardial Jeopardy Score (DMJS) and the QCA-derived SYNTAX Score (SS), both of which are based on invasive angiography [30, 31]. However, these scoring systems were developed based on CAD patients without consideration of the overall impact on the heart when AS is also present. Spatial changes in PCAT attenuation in CCTA images can reflect changes in the entire heart after TAVR. Our study found that PCAT attenuation levels were notably higher in patients who suffered a MACE. The elevated attenuation reflects a heightened inflammatory state within the coronary vasculature, which is detectable and quantifiable through CCTA. After adding PCAT as a predictor, the C-index and IDI of conventional clinical models increased by 0.82 and 55%, respectively. Interestingly, we also found that revascularization in CAD patients does not influence PCAT attenuation. This relationship persisted irrespective of whether patients had undergone revascularization procedures, underscoring the robustness of PCAT as an independent prognostic tool.

Our study has several limitations. Firstly, as this retrospective study derived the PCAT threshold from a single-center cohort and the same study population, the results lack external validation and may overestimate real-world performance. Thus, future multicenter studies with large sample sizes are needed to confirm the generalizability of these findings. Since this study focused on CAD patients post-TAVR, the applicability of our findings to other populations, such as patients with acute coronary syndrome or prior revascularization, remains to be determined. The median follow-up of 1.5 years may not be sufficient to fully capture long-term events, and insufficient time may have elapsed for patients with shorter follow-up to experience late complications. Furthermore, PCAT attenuation was measured three months post-TAVR, thus excluding patients with early events and introducing potential immortal time bias that could overestimate the prognostic value of PCAT. Studies with longer follow-up times are needed to confirm the sustained predictive ability of PCAT post-TAVR.

## 5. Conclusions

In conclusion, the ability of PCAT to serve as a non-invasive, quantifiable marker of coronary inflammation makes it a promising candidate for predicting the outcome of obstructive CAD following TAVR. The sensitivity of PCAT to changes in the inflammatory state and its correlation with clinical outcomes highlight its superiority and potential as a standard component of post-TAVR patient assessment.

## Availability of Data and Materials

The datasets used and analyzed during the current study are available from the corresponding author on reasonable request.
